# Repeated convergent evolution of parthenogenesis in Acariformes (Acari)

**DOI:** 10.1002/ece3.7047

**Published:** 2020-11-20

**Authors:** Patrick Pachl, Matti Uusitalo, Stefan Scheu, Ina Schaefer, Mark Maraun

**Affiliations:** ^1^ JFB Institute of Zoology and Anthropology University of Göttingen Göttingen Germany; ^2^ Zoological Museum Centre for Biodiversity of Turku Turku Finland; ^3^ Centre of Biodiversity and Sustainable Land Use University of Göttingen Göttingen Germany

**Keywords:** backbone, diversification, evolution, mites, Oribatida, phylogeny, sex

## Abstract

The existence of old species‐rich parthenogenetic taxa is a conundrum in evolutionary biology. Such taxa point to ancient parthenogenetic radiations resulting in morphologically distinct species. Ancient parthenogenetic taxa have been proposed to exist in bdelloid rotifers, darwinulid ostracods, and in several taxa of acariform mites (Acariformes, Acari), especially in oribatid mites (Oribatida, Acari). Here, we investigate the diversification of Acariformes and their ancestral mode of reproduction using 18S rRNA. Because parthenogenetic taxa tend to be more frequent in phylogenetically old taxa of Acariformes, we sequenced a wide range of members of this taxon, including early‐derivative taxa of Prostigmata, Astigmata, Endeostigmata, and Oribatida. Ancestral character state reconstruction indicated that (a) Acariformes as well as Oribatida evolved from a sexual ancestor, (b) the primary mode of reproduction during evolution of Acariformes was sexual; however, species‐rich parthenogenetic taxa radiated independently at least four times (in Brachychthonioidea (Oribatida), Enarthronota (Oribatida), and twice in Nothrina (Oribatida), (c) parthenogenesis additionally evolved frequently in species‐poor taxa, for example, *Tectocepheus*, *Oppiella*, *Rostrozetes*, *Limnozetes*, and *Atropacarus*, and (d) sexual reproduction likely re‐evolved at least three times from species‐rich parthenogenetic clusters, in *Crotonia* (Nothrina), in *Mesoplophora/Apoplophora* (Mesoplophoridae, Enarthronota), and in *Sphaerochthonius*/*Prototritia* (Protoplophoridae, Enarthronota). We discuss possible reasons that favored the frequent diversification of parthenogenetic taxa including the continuous long‐term availability of dead organic matter resources as well as generalist feeding of species as indicated by natural variations in stable isotope ratios.

## Graphical abstract

We investigated the radiation of Acariformes and reconstructed their ancestral mode of reproduction using 18S rDNA. We found that (a) Acariformes as well as Oribatida evolved from a sexual ancestor, (b) species‐rich parthenogenetic taxa radiated independently at least four times in Acariformes, (c) parthenogenesis additionally evolved frequently in species‐poor taxa/genera and (d) sexual reproduction likely re‐evolved at least three times from species‐rich parthenogenetic clusters

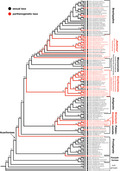

## 1 INTRODUCTION

One of the unsolved enigmas in evolutionary biology is the dominance of sexual reproduction in animal taxa (Brandeis, 2018; Burke & Bonduriansky, 2017; Otto, 2003; Williams, 1975). Despite the many disadvantages of sexual reproduction, including dilution of the genome, breakup of favorable gene combinations, production of males, exposure to predators during courtship and copulation, as well as the transmission of sexual diseases (Bell, 1982; Lehtonen et al., 2013; Maynard Smith, 1978), there must be short‐term advantages of sexual reproduction that prevent the establishment and spread of parthenogenetic lineages (Hartfield & Keightley, 2012). To understand the dominance of sexual reproduction in the animal kingdom, it is instructive to investigate exceptions, that is, animal species that reproduce via parthenogenesis, in particular those persisting for long periods of time (Neiman et al., 2009). Such “ancient asexual scandals” (Maynard Smith, 1978) include bdelloid rotifers, darwinulid ostracods, and several taxa of oribatid mites (Oribatida, Acariformes) (Bode et al., 2010; Brandt et al., 2017; Flot et al., 2013; Ricci, 2017; Schaefer et al., 2010). However, recently it has been shown that bdelloid rotifers engage in some kind of non‐canonical sex (Debortoli et al., 2016) and that there are males in darwinulid ostracods, although very rare (Smith et al., 2006), rendering Oribatida among the last candidates for the evolution and diversification of parthenogenetic taxa (Schwander, 2016). Unfortunately, the phylogeny, evolution, and diversification of Oribatida and Acariformes in general are not well understood, mainly due to the large number of taxa and lineages in this group (Arribas et al., 2019; Maraun et al., 2003, 2004; Pachl et al., 2012; Palmer & Norton, 1991; Schaefer & Caruso, 2019; Schaefer et al., 2010).

Parthenogenetic diversifications are rare events in evolution (Heethoff et al., 2007; Maraun et al., 2004). First, due to the lack of adaptive potential, parthenogenetic taxa have been assumed to be doomed to extinction (Maynard Smith, 1971); however, parthenogenetic taxa may be able to adapt to their environment via phenotypic plasticity and epigenetic mechanisms (Gutekunst et al., 2018). Second, diversification of parthenogenetic lineages appears paradoxical because mechanisms allowing the evolution of morphologically distinct species without sex remain elusive. While sexual taxa are linked via a common gene pool, in parthenogenetic taxa, each lineage evolves independently. Why parthenogenetic Oribatida species form morphologically coherent units therefore remains enigmatic. Rather than morphologically uniform lineages, one may expect a plethora of transition forms to exist in parthenogenetic taxa. However, some species‐rich and monophyletic parthenogenetic taxa, which include morphologically distinct species, likely exist in Oribatida (Domes et al., 2007; Norton & Palmer, 1991; Palmer & Norton, 1991). Those species‐rich monophyletic taxa are unique in the animal kingdom and may therefore essentially contribute to our understanding of the long‐term persistence and diversification of parthenogenetic lineages in animals.

Even more surprising (and possibly more rare) than parthenogenetic diversification is the re‐evolution of sex from parthenogenetic ancestors. There are only a few known instances, one in the plant species *Hieracium pilosella*, the mouse‐ear hawkweed (Asteraceae; Chapman et al., 2003), one in the mite genus *Crotonia* (Oribatida; Domes et al., 2007) and another in ostracods (Horne, 2010). The circumstances that allow or even trigger the re‐evolution of sex are not known, but their cytology may contribute to this pattern. Automictic thelytokous taxa, which still undergo meiosis, may more easily re‐evolve sex than apomicts that lost meiosis entirely. Also, ecological conditions might favor sexual reproduction and therefore the re‐evolution of sex, for example, the transition from plentiful to scarce and heterogeneously distributed resources during evolution (Scheu & Drossel, 2007).

Acariformes, particularly Oribatida, are perfect model organisms to study parthenogenetic radiations and re‐evolution of sex. They include an exceptional high proportion of parthenogenetic taxa, with many of them being phylogenetically old and having radiated in the Carboniferous or earlier (Heethoff et al., 2009; Pachl et al., 2017; Schaefer et al., 2010). Especially the early‐derivative taxa in Oribatida, such as Enarthronota and Nothrina, include many parthenogenetic species, which is surprising because parthenogenesis is often assumed to lack the adaptive potential to persist in the long term (Maynard Smith, 1971, 1978).

Here, we investigated the phylogeny of Acariformes (Prostigmata, Astigmata, Endeostigmata, and Oribatida), with a focus on early‐derivative Oribatida and inferred their ancestral mode of reproduction (sexual vs. parthenogenetic). The phylogenetic relationships among early‐derivative lineages of Oribatida and their monophyly are controversial (Arribas et al., 2019). To independently resolve phylogenetic relationships, we used sequences of 18S rRNA, a gene that allows resolving deep splits in Oribatida (Schaefer et al., 2010) and other Acari; for example, it has been used to unveil the evolution of Parasitiformes (Klompen et al., 2007), the origin and higher‐level diversification of Acariformes (in combination with LSU; Pepato & Klimov, 2015), and the phylogenetic position of the Eriophyoidea within Acariformes (Xue et al., 2017). Importantly, Oribatida may include a wide range of taxa with equivocal phylogenetic position, such as Astigmata and Endeostigmata, and represent the major taxa of Acariformes.

We hypothesized that (a) the ancestral mode of reproduction (i.e., the backbone of the phylogenetic tree) in Acariformes and Oribatida is sexual because the alternative hypothesis is unlikely. If the backbone would have been parthenogenetic, sexual reproduction would have re‐evolved several times, which is very unlikely. If the ancestral mode of reproduction had been sexual, we furthermore hypothesized that (b) parthenogenesis evolved several times independently within Acariformes, and that (c) parthenogenetic taxa radiated into several distinct morphological species. Finally, because few species‐rich parthenogenetic taxa include sexual species we hypothesized that (d) sexual reproduction re‐evolved occasionally in Acariformes.

## 2 MATERIALS AND METHODS

### 2.1 Taxon sampling

In total, 130 species comprising 119 Acariformes (81 Oribatida, 11 Endeostigmata, 13 Astigmata, 14 Prostigmata) and five Parasitiformes, with six non‐Acari Arachnida as outgroup taxa, were included in the dataset. The 81 Oribatida included representatives of the six major phylogenetic groups (26 Brachypylina, 15 Nothrina, 22 Enarthronota, 11 Mixonomata, five Palaeosomata, and two Parhyposomata). Of these, 69 Oribatida were identified at species level and twelve at genus level. Sixteen taxa were newly sampled and sequenced for our study; all other sequences were obtained from NCBI. New species/specimens were extracted by using a heat gradient (Kempson et al., 1963), and mites were determined using relevant taxonomic literature, particularly Balogh and Balogh (1988, 1990, 2002) and Weigmann (2006). Specimens were collected from tropical montane rainforests in southern Ecuador (Illig et al., 2010), temperate forests in central Germany (Erdmann et al., 2012) and various locations in the United States of America, and from several sites all over the world (Table 1). Oribatida species were assigned to higher taxonomic groups according to the classification of Norton and Behan‐Pelletier (2009). Modes of reproduction (i.e., sexual or parthenogenetic) were taken from literature (Maraun et al., 2019; Norton et al., 1993). Mode of reproduction is known from rearing experiments and sex ratios, but in part also was ascribed based on the reproductive mode of sister taxa. Since biological species concepts sensu Mayr (1963) do not apply for parthenogenetic species, we adopted the morphological species concept of Cronquist (1978).

**TABLE 1:** Species name, phylogenetic affiliation, reproductive mode, and GenBank accession numbers of Acariformes, other Chelicerate taxa, and outgroups

Species/genus	Family		Superfamily		Taxon/Supercohort	Reproductive mode (coded for MESQUITE)		GenBank accession nrs. 18S
*Carcinoscorpius rotundicauda* (Pocock, 1902)					Xiphosura (outgroup)	Sexual		HQ588739
*Limulus polyphemus* (Linnaeus, 1758)					Xiphosura (outgroup)	Sexual		L81949
*Chelifer cancroides* (Linnaeus, 1758)					Pseudoscorpiones	Sexual		KT354350
*Ellingsenius indicus* (Chamberlin, 1932)					Pseudoscorpiones	Sexual		KT354353
*Eusimonia wunderlichi* (Kraepelin, 1899)					Solifugae	Sexual		U29492
*Gluvia dorsalis* (C.L. Koch, 1842)					Solifugae	Sexual		AF007103
*Neocarus* sp. (Chamberlin & Mulaik, 1942)					Opilioacarida	Sexual		KP276467
*Opilioacarus texanus* (Chamberlin & Mulak, 1942)					Opilioacarida	Sexual		AF115375
*Amblyomma sphenodonti* (Dumbleton, 1943)					Ixodidae	Sexual		DQ507238
*Pergamasus canestrinii* (Berlese, 1884)	Parasitidae		Parasitoidea		Mesostigmata	Sexual		AY620934
*Trachytes* sp. (Michael, 1894)	Trachytidae		Polyaspidoidea		Mesostigmata	Sexual		MT683118
*Anystis* sp. (Heyden, 1826)	Anystidae		Anystoidea		Trombidiformes	Sexual		KP325052
*Bdellodes* sp. (Oudemans, 1937)	Bdellidae		Bdelloidea		Trombidiformes	Sexual		HM070358
*Spinibdella* sp. (Thor, 1930)	Bdellidae		Bdelloidea		Trombidiformes	Sexual		HM070368
*Microcaeculus* sp. (Franz, 1952)	Caeculidae	Caeculoidea		Trombidiformes			Sexual	AF287232
*Neochelacheles messersmithi* (Smiley & Williams, 1972)	Cheyletidae	Cheyletoidea		Trombidiformes			Sexual	AY620908
*Balaustium* sp. (Von Heyden, 1826)	Erythraeidae	Erythraeoidea		Trombidiformes			Sexual	EF203775
*Eupodes* sp. (Koch, 1835)	Eupodidae	Eupodoidea		Trombidiformes			Sexual	HM070365
*Diplothrombium* sp. (Berlese, 1910)	Johnstonianidae	Trombiculoidea		Trombidiformes			Sexual	KM100930
*Labidostomma* sp. (Kramer, 1879)	Labidostomatidae	Labidostomatoidea		Trombidiformes			Sexual	EF203774
*Tanytydeus* sp. (Theron et al, 1969)	Paratydeidae	Paratydeoidea		Trombidiformes			Sexual	KY922147
*Eotetranychus uchidai* (Ehara, 1956)	Tetranychidae	Tetranychoidea		Trombidiformes			Sexual	AB926274
*Oligonychus rubicundus* (Ehara, 1971)	Tetranychidae	Tetranychoidea		Trombidiformes			Sexual	AB926290
*Tetranychus urticae* (Koch, 1836)	Tetranychidae	Tetranychoidea		Trombidiformes			Sexual	AB926313
*Yezonychus sapporensis* (Ehara, 1978)	Tetranychidae	Tetranychoidea		Trombidiformes			Sexual	AB926258
*Acarus gracilis* (Hughes, 1975)	Acaridae	Acaroidea		Astigmata			Sexual	EF203769
*Aleuroglyphus ovatus* (Troupeau, 1879)	Acaridae	Acaroidea		Astigmata			Sexual	EF203770
*Naiadacarus arboricola* (Fashing, 1974)	Acaridae	Acaroidea		Astigmata			Sexual	JQ000114
*Tyrophagus brevicrinatus* (Robertson, 1959)	Acaridae	Acaroidea		Astigmata			Sexual	MT683111
*Austroglycyphagus (=Glycycometus) geniculatus* (Vitzthum, 1919)	Aeroglyphidae	Glycyphagoidea		Astigmata			Sexual	EF203773
*Arrunsithiana* nr. *spicantis* (Summers and Schuster, 1979)	Canestriniidae	Canestrinioidea		Astigmata			Sexual	JQ000086
*Dermacarus tamiasciuri* (Rupes, Yunker, and Wilson, 1971)	Canestriniidae	Canestrinioidea		Astigmata			Sexual	KP325070
*Carpoglyphus lactis* (Linnaeus, 1767)	Carpoglyphidae	Hemisarcoptoidea		Astigmata			Sexual	EF203772
*Lepidoglyphus destructor* (Schrank, 1781)	Glycyphagidae	Glycyphagoidea		Astigmata			Sexual	EF203771
*Nanacarus* sp. (Oudemans, 1902)	Hemisarcoptidae	Hemisarcoptoidea		Astigmata			Sexual	JQ000068
*Histiostoma feroniarum* (Dufour, 1839)	Histiostomatidae	Histiostomatoidea		Astigmata			Sexual	GQ864328
*Neottialges vitzthumi* (Fain, 1967)	Hypoderatidae	Hypoderatoidea		Astigmata			Sexual	JQ000122
*Dermatophagoides pteronyssinus* (Trouessart, 1898)	Pyroglyphidae	Analgoidea		Astigmata			Sexual	MT683110
*Alicorhagia* sp. (Berlese, 1910)	Alicorhagiidae			Endeostigmata			Parthenogenetic	EU675633
*Alycus* sp. (C. L. Koch, 1842)	Alycidae			Endeostigmata			Sexual	MT683115
*Bimichaelia* sp. (Thor, 1902)	Alycidae			Endeostigmata			Sexual	KY922112
*Pachygnathus* (Dugès, 1834)	Alycidae			Endeostigmata			Sexual	KY922115
*Hybalicus* sp. (Berlese, 1913)	Hybalicidae			Endeostigmata			Parthenogenetic	MT683116
*Micropsammus* sp. (Coineau & Théron, 1983)	Micropsammidae			Endeostigmata			Sexual	KY922132
*Nanorchestes* sp. (Topsent & Trouessart, 1890)	Nanorchestidae			Endeostigmata			Sexual	KP325043
*Cunliffea* sp. (Schubart, 1973)	Nematalycidae			Endeostigmata			Sexual	KY922118
*Gordialycus* sp. (Coineau, Fize and Delamare Deboutteville, 1967)	Nematalycidae			Endeostigmata			Sexual	KY922131
Oehserchestidae sp. (Kethley, 1977)	Terpnacaridae			Endeostigmata			Sexual	KP325049
*Terpnacarus gibbosus* (Womersley, 1944)	Terpnacaridae			Endeostigmata			Parthenogenetic	AY620904
*Stomacarus ligamentifer* (Hammer, 1967)	Archeonothridae	Acaronychoidea		Oribatida/Palaeosomatides			Sexual	EU433992
*Zachvatkinella* sp. (Lange, 1954)	Archeonothridae	Acaronychoidea		Oribatida/Palaeosomatides			Sexual	EF203776
*Beklemishevia galeodula* (Zachvatkin, 1945)	Ctenacaridae	Ctenacaroidea		Oribatida/Palaeosomatides			Sexual	KP325051
*Ctenacarus araneola* (Grandjean, 1932)	Ctenacaridae	Ctenacaroidea		Oribatida/Palaeosomatides			Sexual	EU433991
*Palaeacarus hystricinus* (Trägardh, 1932)	Palaeacaridae	Palaeacaroidea		Oribatida/Palaeosomatides			Parthenogenetic	EF204472
*Atopochthonius artiodactylus* (Grandjean, 1949)	Atopochthoniidae	Atopochthonioidea		Oribatida/Enarthronotides			Parthenogenetic	EU432216
*Brachychthonius bimaculatus* (Willmann, 1936)	Brachychthonidae	Brachychthonioidea		Oribatida/Enarthronotides			Parthenogenetic	MK630360
*Liochthonius peduncularius* (Strenzke, 1951)	Brachychthoniidae	Brachychthonioidea		Oribatida/Enarthronotides			Parthenogenetic	MK630365
*Neoliochthonius piluliferus* (Forsslund, 1942)	Brachychthoniidae	Brachychthonioidea		Oribatida/Enarthronotides			Parthenogenetic	MK630366
*Cosmochthonius lanatus* (Michael, 1885)	Cosmochthoniidae	Protoplophoroidea		Oribatida/Enarthronotides			Sexual	JN585919
*Eniochthonius minutissimus* (Berlese, 1904)	Eniochthoniidae	Hypochthonioidea		Oribatida/Enarthronotides			Parthenogenetic	KR081609
*Haplochthonius simplex* (Willmann, 1930)	Haplochthoniidae	Protoplophoroidea		Oribatida/Enarthronotides			Parthenogenetic	EU675634
*Hypochthonius rufulus* (Koch, 1835)	Hypochthoniidae	Hypochthonioidea		Oribatida/Enarthronotides			Parthenogenetic	KR081618
*Lohmannia banksi* (Norton, Metz & Sharma, 1978)	Lohmanniidae	Hypochthonioidea		Oribatida/Enarthronotides			Parthenogenetic	AF022036
*Meristacarus* sp. (Grandjean, 1934)	Lohmanniidae	Hypochthonioidea		Oribatida/Enarthronotides			Parthenogenetic	KP276478
*Meristolohmannia meristacaroides* (Balogh & Mahunka, 1966)	Lohmanniidae	Hypochthonioidea		Oribatida/Enarthronotides			Parthenogenetic	AY620905
*Mixacarus brevipes* (Banks, 1947)	Lohmanniidae	Hypochthonioidea		Oribatida/Enarthronotides			Parthenogenetic	JN585913
*Nesiacarus granulatus* (Hammer, 1972)	Lohmanniidae	Hypochthonioidea		Oribatida/Enarthronotides			Parthenogenetic	JN585914
*Apoplophora* sp. (Aoki, 1980)	Mesoplophoridae	Hypochthonioidea		Oribatida/Enarthronotides			Sexual	JN585917
*Archoplophora rostralis* (Willmann, 1930)	Mesoplophoridae	Hypochthonioidea		Oribatida/Enarthronotides			Parthenogenetic	JN585918
*Mesoplophora cubana* (Calugar & Vasiliu, 1977)	Mesoplophoridae	Hypochthonioidea		Oribatida/Enarthronotides			Sexual	EU432217
*Nanohystrix hammerae* (Norton & Fuangarworn, 2015)	Nanohystricidae	Heterochthonioidea		Oribatida/Enarthronotides			Sexual	MT683114
*Paralycus* sp. (Womersley, 1944)	Pediculochelidae	Cosmochthonioidea		Oribatida/Enarthronotides			Parthenogenetic	KY922209
*Prototritia major* (Jacot, 1933)	Protoplophoridae	Protoplophoroidea		Oribatida/Enarthronotides			Sexual	JN585915
*Pterochthonius angelus* (Berlese, 1910)	Pterochthoniidae	Atopochthonioidea		Oribatida/Enarthronotides			Parthenogenetic	EU432214
*Sphaerochthonius* sp. (Berlese, 1910)	Sphaerochthoniidae	Protoplophoroidea		Oribatida/Enarthronotides			Sexual	JN585916
*Gozmanyina majestus* (Marshall & Reeves, 1971)	Trichthoniidae	Heterochthonioidea		Oribatida/Enarthronotides			Parthenogenetic	EU433993
*Gehypochtonius urticinus* (Berlese, 1910)	Gehypochthoniidae	Parhypochthonioidea		Oribatida/Parhyposomatides			Parthenogenetic	EU433994
*Parhypochthonius aphidinus* (Berlese, 1904)	Parhypochthoniidae	Parhypochthonioidea		Oribatida/Parhyposomatides			Parthenogenetic	EU432215
*Collohmannia gigantea* (Sellnick, 1922)	Collohmanniidae	Collohmannioidea		Oribatida/Mixonomatides			Sexual	KR081604
*Epilohmannia* sp. (Berlese, 1910)	Epilohmannidae	Epilohmannioidea		Oribatida/Mixonomatides			Sexual	EU432213
*Eulohmannia ribagai* (Berlese, 1910)	Eulohmanniidae	Eulohmannioidea		Oribatida/Mixonomatides			Parthenogenetic	EU432211
*Acrotritia (=Rhysotritia) duplicata* (Grandjean, 1953)	Euphthiracaridae	Euphthiracaroidea		Oribatida/Mixonomatides			Parthenogenetic	EF091417
*Nehypochthonius porosus* (Norton & Metz, 1980)	Nehypochthoniidae	Nehyochthonioidea		Oribatida/Mixonomatides			Parthenogenetic	AF022038
*Indotritia krakatauensis* (Sellnick, 1923)	Oribotritiidae	Euphthiracaroidea		Oribatida/Mixonomatides			Sexual	JN85920
*Perlohmannia* sp. (Berlese, 1916)	Perlohmanniidae	Perlohmannioidea		Oribatida/Mixonomatides			Sexual	EU432212
*Atropacarus striculus* (Koch, 1835)	Phthiracaridae	Phthiracaroidea		Oribatida/Mixonomatides			Parthenogenetic	EF091416
*Phthiracarus* sp. (Perty, 1841)	Phthiracaridae	Phthiracaroidea		Oribatida/Mixonomatides			Sexual	KR081629
*Steganacarus magnus* (Nicolet, 1855)	Phthiracaridae	Phthiracaroidea		Oribatida/Mixonomatides			Sexual	AF022040
*Synichotritia caroli* (Walker, 1965)	Synichotritiidae	Euphthiracaroidea		Oribatida/Mixonomatides			Sexual	MT683117
*Camisia segnis* (Hermann, 1804)	Camisiidae	Crotonioidea		Oribatida/Nothrina			Parthenogenetic	EU432209
*Platynothrus peltifer* (Koch, 1839)	Camisiidae	Crotonioidea		Oribatida/Nothrina			Parthenogenetic	EF091422
*Crotonia brachyrostrum* (Hammer, 1966)	Crotoniidae	Crotonioidea		Oribatida/Nothrina			Sexual	EF081303
*Hermannia gibba* (Koch, 1839)	Hermanniidae	Crotonioidea		Oribatida/Nothrina			Sexual	EF091426
*Trimalaconothrus* sp. (Berlese, 1916)	Malaconothridae	Crotonioidea		Oribatida/Nothrina			Parthenogenetic	EU432210
*Masthermannia* sp. (Berlese, 1913)	Nanhermanniidae	Crotonioidea		Oribatida/Nothrina			Parthenogenetic	KY922217
*Nanhermannia* nana (Nicolet, 1855)	Nanhermanniidae	Crotonioidea		Oribatida/Nothrina			Parthenogenetic	KR081624
*Nothrus truncatus* (Banks, 1895)	Nothridae	Crotonioidea		Oribatida/Nothrina			Parthenogenetic	EF081306
*Novonothrus flagellatus* (Hammer, 1966)	Nothridae	Crotonioidea		Oribatida/Nothrina			Sexual	EF081307
*Afronothrus* sp. (Wallwork, 1961)	Trhypochthoniidae	Crotonioidea		Oribatida/Nothrina			Parthenogenetic	EU152476
*Allonothrus russeolus* (Wallwork, 1960)	Trhypochthoniidae	Crotonioidea		Oribatida/Nothrina			Parthenogenetic	AF022025
*Archegozetes longisetosus* (Aoki, 1965)	Trhypochthoniidae	Crotonioidea		Oribatida/Nothrina			Parthenogenetic	HQ661379
*Mainothrus badius* (Berlese, 1905)	Trhypochthoniidae	Crotonioidea		Oribatida/Nothrina			Parthenogenetic	EF081301
*Mucronothrus nasalis* (Willmann, 1929)	Trhypochthoniidae	Crotonioidea		Oribatida/Nothrina			Parthenogenetic	EF081299
*Trhypochthonius americanus* (Ewing, 1908)	Trhypochthoniidae	Crotonioidea		Oribatida/Nothrina			Parthenogenetic	JQ000046
*Achipteria coleoptrata* (Linnaeus, 1758)	Achipteriidae	Achipterioidea		Oribatida/Brachypylina			Sexual	EF091418
*Caleremaeus monilipes* (Michael, 1882)	Caleremaeidae	Ameroidea		Oribatida/Brachypylina			Sexual	MK630361
*Carabodes subarcticus* (Trägårdh, 1902)	Carabodidae	Carabodoidea		Oribatida/Brachypylina			Sexual	EF091429
*Cepheus dentatus* (Michael, 1888)	Cepheidae	Cepheoidea		Oribatida/Brachypylina			Sexual	MK630354
*Oromurcia sudetica* (Willmann, 1939)	Ceratozetidae	Ceratozetoidea		Oribatida/Brachypylina			Sexual	EU432194
*Scapheremaeus palustris* (Sellnick, 1924)	Cymbaeremaeidae	Cymbaeremaeoidea		Oribatida/Brachypylina			Sexual	EU433989
*Eueremaeus oblongus* (Koch, 1835)	Eremaeidae	Eremaeoidea		Oribatida/Brachypylina			Sexual	EU432205
*Eremaeozetes* sp. (Berlese, 1913)	Eremaeozetidae	Eremaeozetoidea		Oribatida/Brachypylina			Sexual	EU432187
*Galumna lanceata* (Oudemans, 1900)	Galumnidae	Galumnoidea		Oribatida/Brachypylina			Sexual	KX397630
*Gymnodamaeus bicostatus* (Koch, 1835)	Gymnodamaeidae	Plateremaeoidea		Oribatida/Brachypylina			Sexual	KR081614
*Rostrozetes ovulum* (Berlese, 1908)	Haplozetidae	Oripodoidea		Oribatida/Brachypylina			Parthenogenetic	HM070342
*Humerobates rostrolamellatus* (Grandjean, 1936)	Humerobatidae	Ceratozetoidea		Oribatida/Brachypylina			Sexual	EU432196
*Hydrozetes thienemanni* (Strenzke, 1943)	Hydrozetidae	Hydrozetoidea		Oribatida/Brachypylina			Sexual	KX397633
*Xenillus discrepans* (Hermann, 1804)	Liacaridae	Gustavioidea		Oribatida/Brachypylina			Sexual	EU432203
*Limnozetes foveolatus* (Willmann, 1939)	Limnozetidae	Hydrozetoidea		Oribatida/Brachypylina			Parthenogenetic	KX397634
*Mycobates tridactylus* (Willmann, 1929)	Mycobatidae	Ceratozetoidea		Oribatida/Brachypylina			Sexual	MT683112
*Poroliodes farinosus* (Koch, 1839)	Neoliodidae	Neoliodioidea		Oribatida/Brachypylina			Sexual	EF203779
*Oppiella nova* (Oudemans, 1902)	Oppiidae	Oppioidea		Oribatida/Brachypylina			Parthenogenetic	KR081626
*Liebstadia humerata* (Sellnick, 1928)	Oribatulidae	Oripodoidea		Oribatida/Brachypylina			Sexual	KR081620
*Oribatula tibialis* (Nicolet, 1855)	Oribatulidae	Oripodoidea		Oribatida/Brachypylina			Sexual	EU433990
*Ceratoppia bipilis* (Hermann, 1804)	Peloppiidae	Gustavioidea		Oribatida/Brachypylina			Sexual	EU432204
*Eupelops plicatus* (Koch, 1835)	Phenopelopidae	Phenopelopoidea		Oribatida/Brachypylina			Sexual	EF091419
*Scheloribates ascendens* (Weigmann & Wunderle, 1990)	Scheloribatidae	Oripodoidea		Oribatida/Brachypylina			Sexual	MT683113
*Tectocepheus sarekensis* (Trägårdh, 1910)	Tectocepheidae	Tectocepheoidea		Oribatida/Brachypylina			Parthenogenetic	EF093776
*Hafenrefferia gilvipes* (Koch, 1839)	Tenuialidae	Gustavioidea		Oribatida/Brachypylina			Sexual	MK630363
*Banksinoma lanceolata* (Michael, 1885)	Thyrisomidae	Oppioidea		Oribatida/Brachypylina			Sexual	MK630359

### 2.2 DNA extraction and PCR

Genomic DNA was extracted from single individuals using the DNeasy Blood and Tissue Kit (Qiagen) with silica membrane columns and protease K from Genaxxon (25 mM; Genaxxon BioScience). Amplification of target genes was performed in 25 µl volume. Primers for 18S rDNA were 5′‐TAC CTG GTT GAT CCT GCC AG‐3′ (18Sforward) and 5′‐AAT GAT CCT TCC GCA GGT TCA C‐3′ (18Sreverse) (Domes et al., 2007). The 18S rDNA fragment was amplified at 57°C using standard PCR protocols. PCR products were sequenced at Göttingen Genomics Laboratory (Institute of Microbiology and Genetics, University of Göttingen, Germany), using the additional sequencing primers 18S554f 5′‐AAG TCT GGT GCC AGC AGC CGC‐3′, 18S1282r 5′‐TCA CTC CAC CAA CTA AGA ACG GC‐3′, 18S1150f 5′‐ATT GAC GGA AGG GCA CCA CCA G‐3′ and 18S614r 5′‐TCC AAC TAC GAG CTT TTT AAC C‐3′ (Domes et al., 2007). Sequences MK630354, MK630359‐61, MK630363, MK630365‐66, and MT683110‐18 were generated for this study (in total 16 sequences; Table 1); all other sequences were obtained from NCBI. We used the 18S rRNA gene since no other reliable genes for reconstructing the phylogeny of Acariformes are available.

### 2.3 Sequence alignment and phylogenetic analysis

The 18S rDNA gene sequences generated for this study were assembled and edited in Sequencher 5.1 (Gene Codes Corporation); ambiguous positions were corrected using the chromatograms. The final alignment of 130 sequences had a total length of 2,455 characters; the shortest sequence had 1,468 bp (*Pergamasus canestrinii*, Mesostigmata), the longest 1,897 bp (*Haplochthonius simplex*, Enarthronota). Sequences of the alignment were trimmed to the shortest sequence downloaded from NCBI. Sequences were aligned in ClustalX 2.1 (Larkin et al., 2007). Several gap opening and gap extension parameters were tested and used for Maximum‐Likelihood reconstruction in R v3.6 (R Core Team, 2018) using the *pml* function of the *phangorn* package (Schliep, 2011). The parameters for gap opening = 20 and gap extension = 0.1 resulted in the best‐supported phylogeny. Partitioning of sequences into conserved and variable regions and applying different alignment parameters and models of sequence evolution did not improve the phylogenetic trees or bootstraps. The final phylogenetic tree was constructed with IQ‐TREE v1.6 (Nguyen et al., 2015) using ModelFinder (Kalyaanamoorthy et al., 2017) and ultrafast bootstrap (Hoang et al., 2018) with 1,000 bootstrap replicates and setting *Limulus polyphemus* (Xiphosura) as outgroup taxon. To test for the robustness of the ML tree, we also calculated trees in MrBayes using the same settings (lset nst = 6 rates = gammainv) and the same outgroup. We ran the mcmc chain for 5 million generations, with a sample frequency of 5,000 and a burnin of 25%. The resulting tree had the same topology as the ML tree, except that nodes with lower bootstrap support were not resolved and were displayed as polytomies in the Bayesian tree (Figure S1).

### 2.4 Inferring the ancestral reproductive mode

We used Mesquite 3.61 (Maddison & Maddison, 2019) to map the mode of reproduction as a character on the phylogenetic tree. Character history was traced using parsimony to infer patterns of the ancestral state of reproduction using the symmetrical Mk1 (Markov *k‐*state 1 parameter) model using the parameters 1, 5, and 10 for character change and asymmetrical models with higher rates for the loss of sex (5:1, 10:1). The ancestral character history did not differ among these models. Likelihood analyses using the same parameter as for the asymmetrical model resulted in fully ambiguous backbone. Because reproductive modes are complex traits, we continue with the results of the parsimony inference. The Maximum‐Likelihood tree generated by IQ‐Tree provided the topology, and the present‐day reproductive mode of investigated species was coded as sexual or parthenogenetic.

## 3 RESULTS

### 3.1 Ancestral mode of reproduction and reproductive mode during evolution

**FIGURE 1:**
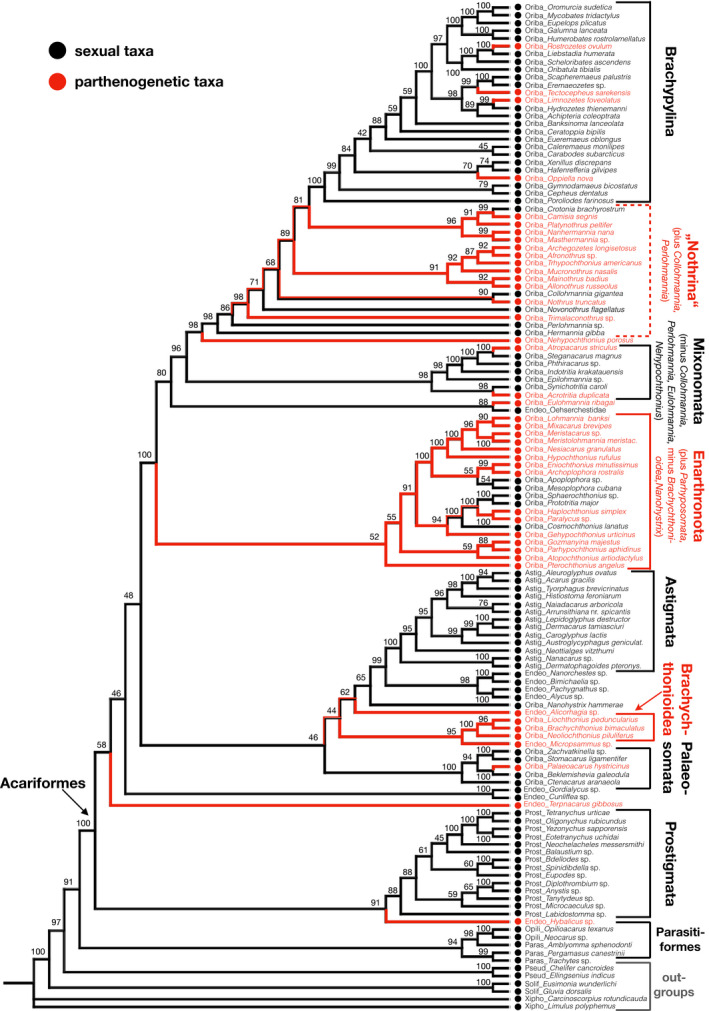
Maximum‐likelihood tree of Acariformes based on 18S rRNA gene of Acariformes including three other arachnid taxa (Parasitiformes, Pseudoscorpiones, Solifugae) and two outgroup taxa (Xiphosura). Numbers at nodes represent bootstrap supports (1,000 replicates). Ancestral character state reconstruction of the reproductive mode (sexual = black, parthenogenetic = red) was carried out using Maximum Parsimony in Mesquite (Maddison &amp; Maddison, 2019). Oriba: Oribatida, Endeo: Endostigmata, Prost: Prostigmata, Opili: Opilioacariformes, Paras: Parasitiformes, Pseud: Pseudoscorpiones, Solif: Solifugae, Xipho: Xiphosura

Ancestral character state reconstruction indicated that the plesiomorphic state of reproduction in Acariformes and in Oribatida was sexual (Figure 1). Outgroup taxa (mainly Prostigmata) were mainly sexual. In general, Endeostigmata clustered at separate positions in the phylogenetic tree, suggesting that the taxon is not monophyletic. Ancestral character state reconstruction further indicated that the mode of reproduction of Oribatida during evolution was mainly sexual; however, some remained uncertainty at intermediate positions of the backbone (Figure 1).

### 3.2 Convergent evolution of parthenogenesis

According to the ancestral character state reconstruction (Figure 1), the sampled species represent at least 17 independent evolutionary events of thelytoky. The number, however, remains ambiguous as the character state reconstruction of the mode of reproduction in some cases was only weakly supported. Notably, lineages that switched to thelytoky included lineages with only one or few species, such as *Limnozetes*, *Tectocepheus*, *Rostrozetes*, and *Oppiella nova* in Brachypylina, *Nehypochthonius porosus*, *Atropacarus*, and *Acrotritia* in Mixonomata, *Haplochthonius* in Enarthronota, and *Palaeacarus* in Palaeosomata, but also species‐rich clades, such as Brachychthonioidea, Nothrina, and Enarthronota.

### 3.3 Re‐evolution of sex

Ancestral character state reconstruction indicated that there are three independent cases of re‐evolution of sex in the examined Oribatida, that is, in *Crotonia brachyrostrum* (Nothrina), in *Mesoplophora*/*Apoplophora* (Mesoplophoridae, Enarthronota), and in *Sphaerochthonius*/*Prototritia* (Protoplophoridae, Enarthronota) (Figure 1). Two of these cases were strongly supported by high bootstrap values (99 for *Crotonia brachyrostrum*, 100 for *Sphaerochthonius*/*Prototritia*), one was weakly supported (54 for *Mesoplophora*/*Apoplophora*; Figure 1).

## 4 DISCUSSION

The oribatid mite phylogenetic tree based on the 18S rRNA gene was generally well‐resolved and supported by moderate to high bootstrap values. Additionally, many of its monophyletic taxa (as indicated in Figure 1) agree with morphologically well‐supported taxa (Dabert et al., 2010; Norton & Behan‐Pelletier, 2009; Schaefer et al., 2010; Schäffer et al., 2010; Weigmann, 2006). Taxa which were assumed to be paraphyletic based on morphological characters (e.g., Nothrina, Mixonomata) were also not monophyletic in the tree. Oribatida were also not monophyletic but included Astigmata and several species of Endeostigmata (Norton, 1994; O'Connor, 1984). The generally high bootstrap values, and the close matching with morphologically based evidence of monophyletic and paraphyletic groupings support the overall validity of the tree for inferring the ancestral state of reproduction of Acariformes. However, we are aware that a single locus may not allow to accurately represent the phylogeny of Acariformes and that uncertainties remain about the assignment of the reproductive mode at weakly supported nodes; information on more genes is needed to resolve the phylogeny of Acariformes in particular at ambiguous nodes.

### 4.1 Ancestral mode of reproduction

The phylogenetic tree based on 18S rDNA indicates that the ancestral reproductive mode in Acariformes and in Oribatida is sexual. Astigmata also are exclusively sexual and presumably form a monophyletic clade within Oribatida (Klimov & O'Connor, 2013; Pepato & Klimov, 2015). However, the sister taxon of Astigmata still is unclear, because its phylogenetic position in molecular studies was sensitive to taxon sampling and markers (Dabert et al., 2010; Domes et al., 2007; Klimov & O'Connor, 2013; Pepato & Klimov, 2015). In this study, Astigmata derived with weak support within Brachychthoniidae. Genome data of mites may eventually solve the phylogenetic position of Astigmata among Acariformes. Overall, the many parthenogenetic taxa in Acariformes and in Oribatida with their scattered distribution in the phylogenetic tree support the hypothesis that sex was lost many times during the evolution of Acariformes. Parthenogenetic taxa near the base of the Acariformes phylogeny, for example,. *Alicorhagia* sp. or *Terpnacarus gibbosus*, presumably represent offshoots that evolved parthenogenesis independently. Additionally, the early‐derivative taxa in Oribatida, that is, Palaeosomata, were also sexual further supporting the view that the ancestral reproductive mode in Oribatida was sexual. Overall, our findings indicate that parthenogenesis evolved not at the beginning but later during the evolution of Oribatida.

### 4.2 Reproductive mode of the backbone

The most parsimonious explanation of the ancestral character state reconstruction of the reproductive mode in Acariformes and Oribatida is that their ancestral mode of reproduction was sexual, indicating that this mode of reproduction was maintained throughout their evolution. However, the ancestral character state reconstruction remains somewhat ambiguous due to many parthenogenetic lineages in the tree. Considering that there are only few cases of the re‐evolution of sex (Chapman et al., 2003; Domes et al., 2007; Horne, 2010), whereas parthenogenesis presumably evolved thousands of times (Bell, 1982; Neiman et al., 2014), a parthenogenetic backbone is very unlikely.

### 4.3 Multiple origin and diversification of parthenogenetic taxa

Results of our study confirmed earlier conclusions that parthenogenesis in Oribatida evolved multiple times (Krause et al., 2016; Norton & Palmer, 1991; Pachl et al., 2012, 2017). This supports the suggestion that sex may be lost easily during evolution (Simon et al., 2003), although the routes to parthenogenesis from sexual ancestors are manifold (Bell, 1982; Suomalainen et al., 1987). The frequent and convergent transition from sexual to parthenogenetic reproduction in Oribatida thus may not be surprising; it also occurred, for example, in the lepidopteran species *Dahlica triquetrella* (Elzinga et al., 2013), in the lizard genus *Leiolepis* (Grismer et al., 2014), in the ostracod species *Eucypris virens* (Bode et al., 2010), in grasshopper and gecko species (Kearney et al., 2006), and in *Timema* stick insects (Schwander & Crespi, 2009). However, the benefits of the loss of sex are controversially discussed and include hybrid vigor (=heterosis) (Vrijenhoek, 1998), extension of the geographical range associated with a “general purpose genotype” (Lynch, 1984), enhanced survival of harsh environmental conditions (Kearney et al., 2006), or faster exploitation of unlimited resources (Scheu & Drossel, 2007). For Oribatida, the latter possibly plays a major role because high densities of Oribatida (an indication that resources are plentiful) correlate with high frequency of parthenogenetic species and individuals (Maraun et al., 2012, 2019).

Despite the evolutionary benefits of parthenogenesis, diversification of a parthenogenetic lineage into morphologically distinct species is enigmatic and perhaps unique for Oribatida. The phylogeny of Oribatida presented in this study supports earlier views (Heethoff et al., 2009; Palmer & Norton, 1991) that this happened at least four times, that is, in Brachychthonioidea, Enarthronota, and twice in Nothrina. As this is unique in the animal kingdom (see Tucker et al., 2013), studying Oribatida is most promising for understanding evolutionary consequences of the loss of sex (Heethoff et al., 2007; Palmer & Norton, 1992; Schwander et al., 2014). The diversification of these taxa into different taxonomically recognized species indicates that they successfully split into morphologically coherent units without engaging in sexual processes. However, it remains to be shown whether these diversifications were adaptive or not (Gittenberger, 1991; Schluter, 2000).

Remarkably, the four species‐rich parthenogenetic clusters of Oribatida are very old and likely originated 400–300 mya in Devonian, Carboniferous, and Permian times (Schaefer & Caruso, 2019; Schaefer et al., 2010). In contrast to the commonly held view that parthenogenetic lineages are short lived, it is increasingly realized that there are a number of old asexual taxa (Neiman et al., 2009). Recent studies in Oribatida have shown that parthenogenetic lineages manage to overcome the problems of the accumulation of deleterious mutations, possibly due to strong purifying selection related to large population size (Brandt et al., 2017). The diversification of the four clusters of parthenogenetic species in Oribatida coincides with the massive global carbon burial during the Permian/Carboniferous time (Berner, 2003) providing large amounts of resources for decomposers, presumably resulting in massive population growth favoring parthenogenetic reproduction and potentially diversification of parthenogenetic species (Scheu & Drossel, 2007). Notably, the parthenogenetic lineages survived the large mass extinction events during and at the end of the Paleozoic, potentially also due to large amounts of accumulated dead organic material (Benton & Twitchett, 2003).

Many of the Oribatida taxa that underwent parthenogenetic diversifications still occur today in habitats that resemble dominant ecosystems during Carboniferous times, for example, in boreal forests (i.e., Brachychthonioidea; Maraun & Scheu, 2000), in wet temperate forests (Nothrina), in peat bogs (Nothrina, Enarthronota; Lehmitz & Maraun, 2016), and generally in wet or aquatic habitats (Nothrina) suggesting that these habitats favor parthenogenetic species (Seniczak et al., 2016). High densities of Oribatida in these habitats (up to 200,000 ind./m^2^; Maraun & Scheu, 2000) further support the view that ample resource availability (as indicated by high densities) favors parthenogenetic reproduction. Overall, our findings suggest that ecological factors fostered the evolution of parthenogenesis, its long‐term maintenance, and its subsequent diversification into morphologically coherent units/species.

### 4.4 Re‐evolution of sex

The re‐evolution of complex characters during evolution contradicts Dollo's law (Gould, 1970) stating that complex characters once lost do not re‐evolve (Collin & Miglietta, 2008). Sexual reproduction is such a complex character that presumably evolved only once very early during eukaryote evolution (Bernstein et al., 1984). Once lost its subsequent re‐evolution therefore is unlikely (Bull & Charnov, 1985). However, there are a few cases where sexual reproduction likely has re‐evolved, once in the plant genus *Hieracium* (Chapman et al., 2003), in ostracods (Horne, 2010), and in Oribatida in the taxon Crotoniidae/Camisiidae (Domes et al., 2007). However, there is evidence from our study that in Oribatida sex also re‐evolved twice in Enarthronota. Re‐evolution of sex in Oribatida may have been facilitated by parthenogenetic species reproducing via automixis still undergoing meiosis (Bergmann et al., 2018). The occasional production of spanandric (i.e., very rare) males (Taberly, 1987a, 1987b, 1987c) indicates that they never lost the ability to produce males.

Understanding the driving factors for the re‐evolution of sex is difficult. An interesting pattern related to the re‐evolution of sex is that the species/lineages which re‐evolved sex either are tropical but originated in temperate regions (Pachl et al., 2017) or still live in temperate regions (*Crotonia brachyrostrum*, *Apoplophora* sp., *Mesoplophora cubana*). Oribatida communities in the temperate or boreal zone generally include more parthenogenetic taxa than those in the tropics (Maraun et al., 2019; Pachl et al., 2017). Possibly, the more abundant parthenogenetic species/lineages in temperate and boreal habitats provided more opportunities for the transition to sex. However, the factors which drove these transitions remain enigmatic but might be related to more scarce and more patchily distributed resources (Scheu & Drossel, 2007; Song et al., 2011).

## CONFLICT OF INTEREST

The authors have no conflict of interest.

## AUTHOR CONTRIBUTION

**Patrick Pachl:** Conceptualization (equal); Data curation (equal); Formal analysis (equal); Methodology (equal); Software (equal). **Matti Uusitalo:** Data curation (equal); Validation (equal). **Stefan Scheu:** Conceptualization (equal); Formal analysis (equal); Writing‐review & editing (equal). **Ina Schaefer:** Conceptualization (equal); Data curation (equal); Formal analysis (equal); Investigation (equal); Software (equal); Supervision (equal); Visualization (equal); Writing‐review & editing (equal). **Mark Maraun:** Conceptualization (equal); Formal analysis (equal); Investigation (equal); Project administration (equal); Supervision (equal); Validation (equal); Visualization (equal); Writing‐original draft (equal); Writing‐review & editing (equal).

## Supporting information

Fig S1

## Data Availability

All data are published in NCBI (for GenBank accession numbers see Table 1).
