# Supplementary material for: Repeated convergent evolution of parthenogenesis in Acariformes (Acari)
Source: Ecol Evol. 2020 Nov 20;11(1):321–37. doi: 10.1002/ece3.7047 (PMC7790623; doi:10.1002/ece3.7047)
Supplement: Supplementary file 1 — Fig S1 [file ECE3-11-321-s001.pdf]

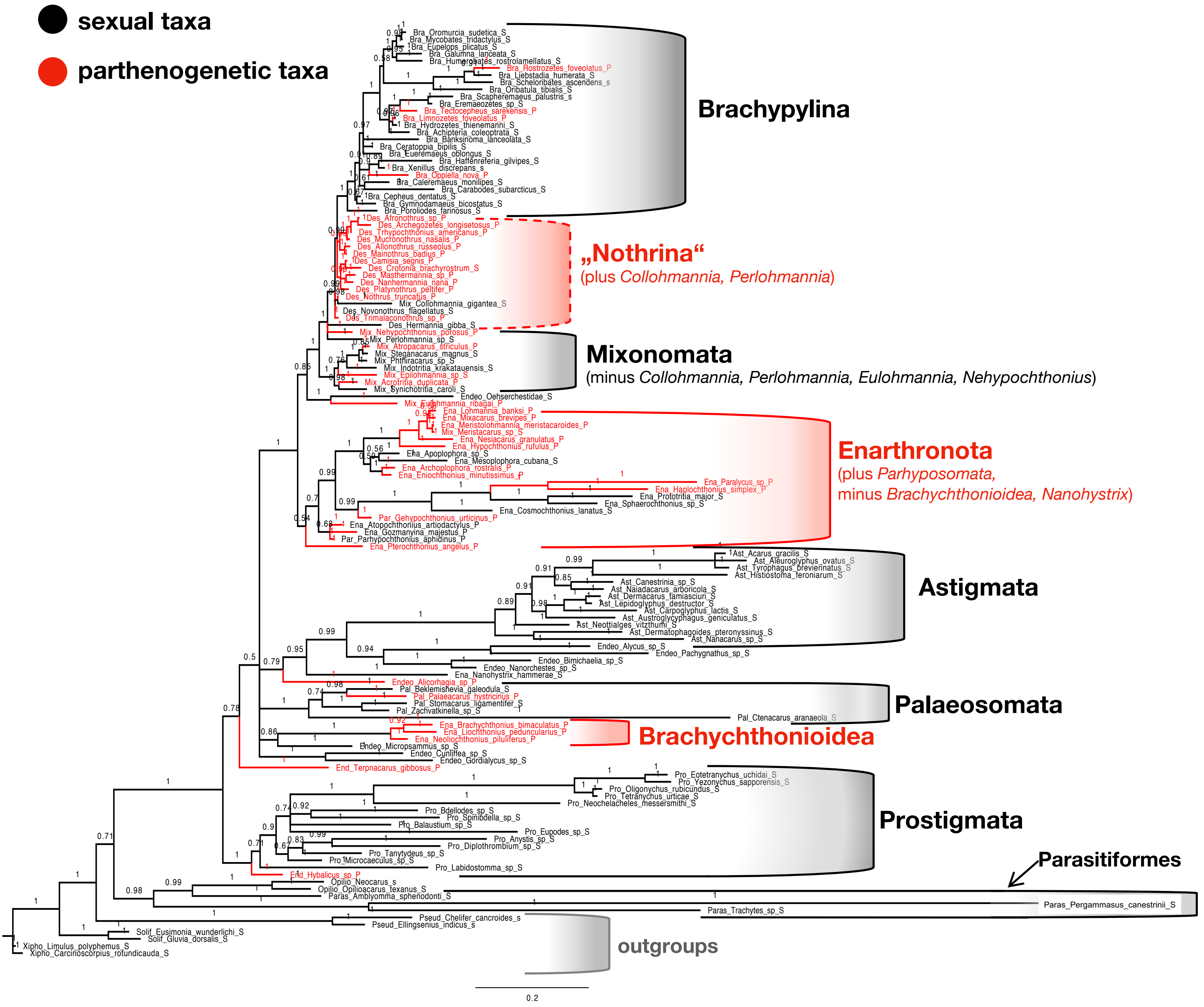
sexual taxa
parthenogenetic taxa
Brachypylina
„Nothrina“
(plus Collohmannia, Perlohmannia)
Mixonomata
(minus Collohmannia, Perlohmannia, Eulohmannia, Nehypochthonius)
Enarthronota
(plus Parhyposomata,
minus Brachychthonioidea, Nanohystrix)
Astigmata
Palaeosomata
Brachychthonioidea
Prostigmata
Parasitiformes
outgroups
0.2
